# A GLP1 receptor agonist diabetes drug ameliorates neurodegeneration in a mouse model of infantile neurometabolic disease

**DOI:** 10.1038/s41598-022-17338-1

**Published:** 2022-08-15

**Authors:** L. Poupon-Bejuit, M. P. Hughes, W. Liu, A. Geard, N. Faour-Slika, S. Whaler, G. Massaro, A. A. Rahim

**Affiliations:** UCL School of Pharmacy, University College London, London, UK

**Keywords:** Neurodegeneration, Neonatal brain damage, Translational research

## Abstract

Infantile neuroaxonal dystrophy (INAD) is a rare paediatric neurodegenerative condition caused by mutations in the *PLA2G6* gene, which is also the causative gene for PARK14-linked young adult-onset dystonia parkinsonism. INAD patients usually die within their first decade of life, and there are currently no effective treatments available. GLP1 receptor (GLP-1R) agonists are licensed for treating type 2 diabetes mellitus but have also demonstrated neuroprotective properties in a clinical trial for Parkinson’s disease. Therefore, we evaluated the therapeutic efficacy of a new recently licensed GLP-1R agonist diabetes drug in a mouse model of INAD. Systemically administered high-dose semaglutide delivered weekly to juvenile INAD mice improved locomotor function and extended the lifespan. An investigation into the mechanisms underlying these therapeutic effects revealed that semaglutide significantly increased levels of key neuroprotective molecules while decreasing those involved in pro-neurodegenerative pathways. The expression of mediators in both the apoptotic and necroptotic pathways were also significantly reduced in semaglutide treated mice. A reduction of neuronal loss and neuroinflammation was observed. Finally, there was no obvious inflammatory response in wild-type mice associated with the repeated high doses of semaglutide used in this study.

## Introduction

Rare infantile neurometabolic diseases are often devastating and early lethal genetic conditions for which there is frequently no effective treatment available. Infantile neuroaxonal dystrophy (INAD, OMIM #256600, also known as neurodegeneration with brain iron accumulation 2A [NBIA2A]) is an intractable neurometabolic infantile disease associated with mutations in the *PLA2G6* gene^1^. *PLA2G6* encodes VIA-calcium independent phospholipase A2 (iPLA2β), a protein that is essential for membrane remodelling in neurons and has variable enzymatic activity depending on the phospholipid composition of these membranes. However, it is unclear how *PLA2G6* mutations lead to the INAD disease phenotype. Mutations in the *PLA2G6* gene also cause the recessive familial type 14 of Parkinson disease (PARK14)^2^, a form of dystonia-parkinsonism characterised by early-onset L-dopa-responsive parkinsonism and cognitive deterioration^3^. While several missense mutations have been linked to PARK14, the exact role of *PLA2G6* in parkinsonism-related disorders is not fully understood. INAD is characterised by neurodegeneration in the nervous system, including abnormalities of nerve endings within the brain, spinal cord, and peripheral nerves. INAD is characterised by early symptoms onset between 6 months and 2 years of age with progressive motor and sensory impairment, which is followed by the loss of ambulation within 5 years^4,5^. A hallmark in disease diagnosis is the presence of axonal spheroids in peripheral nerve biopsies^6^, and patients can present with cerebellar atrophy, with or without iron accumulation^1^. There is currently no approved curative or effective therapy for INAD and treatment options rely on symptomatic relief and palliative care.

Glucagon-like peptide-1 (GLP-1) receptor (GLP-1R) agonists are a group of drugs licensed for the treatment of type 2 diabetes mellitus (T2DM). However, they have also recently been identified as having neuroprotective properties in mouse models of adult neurodegenerative diseases including Alzheimer disease^7^, Parkinson disease^8^ and brain injury^9,10^. Exendin-4 is one of the most studied GLP-1R agonists demonstrating therapeutic efficacy in preclinical studies and a recent clinical trial for PD^11–13^. This research is progressing to a phase 3 clinical trial of this approach in Parkinson Disease patients (ClinicalTrials.gov Identifier: NCT04232969). However, a limitation of exendin-4 is its short half-life of approximately 60–90 min^14^. Therefore, a new generation of GLP-1R agonists have now been developed with significantly longer half-lives. Semaglutide (Ozempic®) is a modified version of another existing GLP-1R agonist, liraglutide, that possesses protease-resistance as a result of an amino acid change at position 8. Subsequently, semaglutide is being marketed as a new once-weekly drug to treat T2DM owing to its longer half-life of approximately 7 days^15^. It has been approved in the USA, Canada and the EU as a treatment for diabetes^16,17^. Semaglutide has shown a similar safety profile to the other GLP-1R agonists currently used for the management of T2DM^18^. Interestingly, it has also demonstrated neuroprotective properties in two pre-clinical studies of Parkinson’s disease^19,20^. These neuroprotective and anti-inflammatory properties are currently being investigated in an ongoing clinical trial for idiopathic Parkinson’s disease (ClinicalTrials.gov identifier: NCT03659682). Here, we investigated for the first time the therapeutic efficacy of the GLP-1R agonist semaglutide in a mouse model of INAD.

## Results

### Semaglutide treatment extends lifespan and rescues locomotor functions in a mouse model of INAD

**Figure 1:**
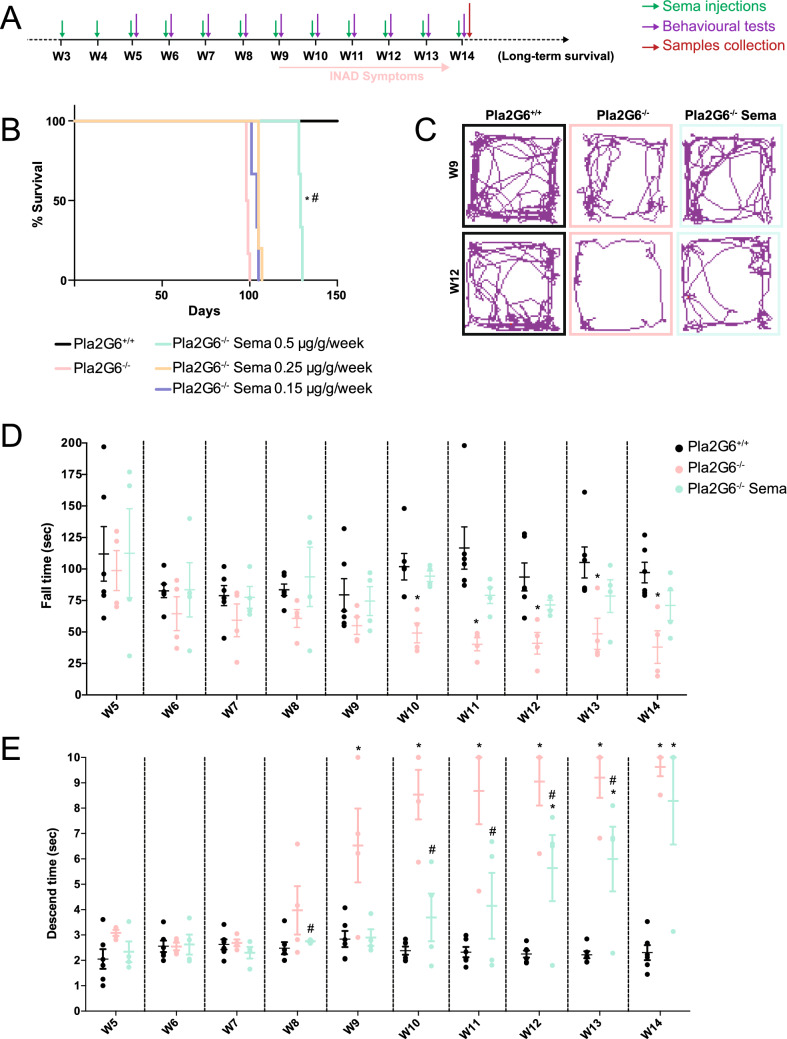
Improvement of survival and locomotor symptoms in *Pla2g6*^−/−^ mice following high-dose weekly semaglutide treatment. (**A**) Schematic of study design. *Pla2g6*^−/−^ mice received weekly IP administration of semaglutide (*n* = 6 per group, 0.5 µg/g, 0.25 μg/g or 0.15 μg/g) starting at 3 weeks old. Behavioural assays were performed for the high-dose treated group (0.5 μg/g/week) every week from week 5 to week 14. Tissue was collected at week 14. (**B**) Survival curve (*n* = 6 per experimental group); (**C**) examples of open field traces at weeks 9 and 12. All data are presented as mean (SEM); ordinary two-way ANOVA adjusted using Dunnett’s multiple comparisons test; *p* values reported in Table S4. (**D**) rotarod performance represented by latency to fall from accelerating rotarod (WT *n* = 6, *Pla2g6*^−/−^* n* = 4; *Pla2g6*−/− Sema *n* = 4), and (**E**) time taken for mice to descend on the pole test (WT *n* = 6, *Pla2g6*^−/−^* n* = 4; *Pla2g6*^−/−^ Sema *n* = 4). * indicates statistically significant difference between the relevant experimental group and WT controls; # indicates statistically significant difference between *Pla2g6*^−/−^ Sema mice and untreated *Pla2g6*^−/−^ controls.

The *Pla2g6-inad* knock-in mouse model (*Pla2g6*^−/−^) was administered with semaglutide at three doses; 0.5 µg/g, 0.25 µg/g or 0.15 µg/g, via intraperitoneal injection once a week. Treatment started from 3 weeks of age and mice were monitored for survival in comparison to age-matched untreated *Pla2g6*^−/−^ mice and wild-type mice (WT) which were included as controls (*n* = 6 per experimental group). The experimental design is outlined in Fig. 1A.

There was no significant increase in survival for *Pla2g6*^−/−^ mice treated with either 0.25 µg/g or 0.15 µg/g doses. A significant increase in survival was observed in *Pla2g6*^−/−^ mice treated with 0.5 µg/g semaglutide, with median survival at 129 days vs 99 days for untreated *Pla2g6*^−/−^ mice (Fig. 1B) (all p-values are shown in Supplementary Table S4). Mice treated with 0.5 µg/g semaglutide were therefore further investigated for improvements in locomotor function from week 5 to week 14 (*n* = 6 for WT; *n* = 4 for untreated *Pla2g6*−/− mice; *n* = 4 for treated *Pla2g6*^−/−^ mice with 0.5 µg/g semaglutide). Assessments were conducted at weekly intervals. Motor function was assessed using a rotarod, where significant progressive deficits were detected in untreated *Pla2g6*^−/−^ mice from 10 weeks of age compared to age-matched wild-type controls until 14 weeks old (Fig. 1D) (all *p*-values are shown in Supplementary Table S4). *Pla2g6*^−/−^ mice treated with 0.5 µg/g semaglutide did not show a significant deficit in rotarod performance compared to wild-type controls at any time point measured.

The vertical pole test was also used to assess locomotor function and hind limb strength. Significant progressive deficits in untreated *Pla2g6*−/− mice were measured as early as 9 weeks of age (Fig. 1D) (all *p*-values are shown in Supplementary Table S4). Treatment of *Pla2g6*^−/−^ mice with 0.5 µg/g semaglutide resulted in a significant improvement in performance of the vertical pole test compared to untreated mice from week 9 to week 13; however, the performances of the two groups were not statistically significantly different at 14 weeks when the humane end-point for untreated mice was reached. The open field test (Fig. 1C) in untreated *Pla2g6*^−/−^ mice revealed abnormalities in mobility in all the measured parameters, excluding maximum speed, at 12 weeks old in comparison to WT controls (Fig. S1). Data analyses showed a significant reduction in the total distance travelled, average speed, total mobility time, total immobility time and freezing time measurements in 12-week-old *Pla2g6*^−/−^ mice when compared to wild-type counterparts. Data analyses didn’t reveal a significant difference when comparing the untreated *Pla2g6*^−/−^ mice to the *Pla2g6*^−/−^ group treated with high-dose semaglutide, except for a significant decrease in the freezing time at 12 weeks. The *Pla2g6*^−/−^ group treated with high-dose semaglutide were comparable to age-matched wild type controls for all measured parameters, excluding the total mobility and immobility time.

### Activation of the GLP-1 receptor promotes neuroprotective pathways and reduces neurodegeneration

**Figure 2:**
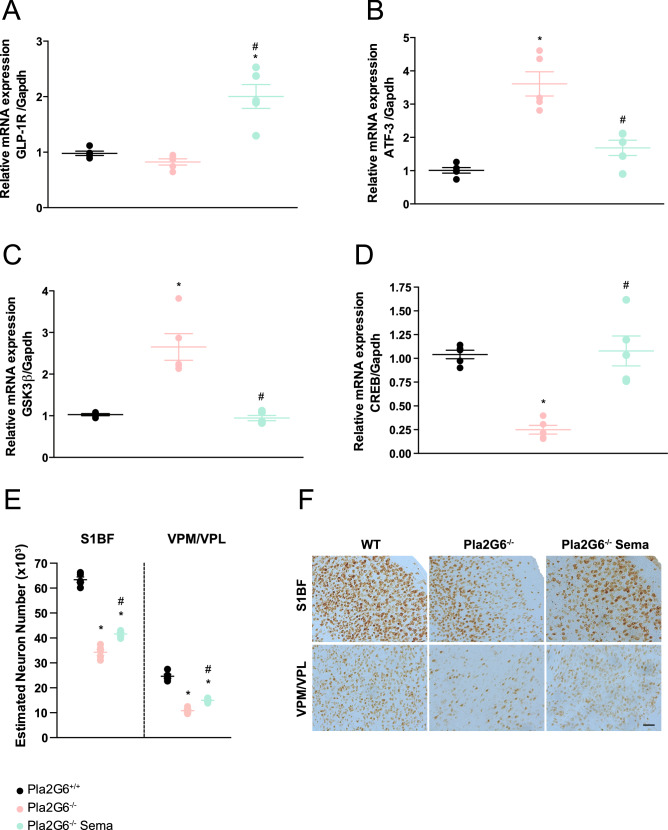
Semaglutide induces neuroprotection and reduces neuronal damage demonstrated by modulation of neuroprotective markers and neuronal damage markers. Relative quantification of (**A**) GLP-1R, (**B**) ATF-3, (**C**) GSK3β and (**D**) CREB, with Gapdh as internal reference by quantitative PCR. RT-qPCR performed from RNA samples prepared from WT, untreated *Pla2g6*^−/−^ and treated *Pla2g6*^−/−^ Sema brain samples (*n* = 5 per group). (**E**) Optical fractionator estimation of NeuN stained neuronal numbers in the somatosensory barrel-field cortex (S1BF) and ventral posteromedial nucleus/ventral posterolateral nucleus (VPM/VPL) of WT, untreated *Pla2g6*^−/−^ and treated *Pla2g6*^−/−^ (*n* = 5 per group) brain sections. (**F**) Representative images of brain sections stained with NeuN. Scale bar = 100 µm. All data are presented as mean (SEM); ordinary one-way ANOVA adjusted using Tukey’s multiple comparisons test; *p* values reported in Table S5. * indicates statistically significant difference between the relevant experimental group and WT controls; # indicates statistically significant difference between *Pla2g6*^−/−^ Sema mice and untreated *Pla2g6*^−/−^ controls.

We next examined the ability of semaglutide to induce neuroprotection by analysis of different factors known to be involved in neurodegenerative diseases. We first looked at the levels of GLP-1R expression in comparison with wild-type mice and untreated controls using qPCR. A significant increase in GLP-1R expression was observed in *Pla2g6*^−/−^ mice treated with 0.5 µg/g semaglutide at 14 weeks when compared to age-matched wild-type and untreated *Pla2g6*^−/−^ controls (Fig. 2A) (all *p*-values are shown in Supplementary Table S5).

Activating transcription factor 3 (ATF-3) is induced in various tissues in response to stress and is used as a neuronal damage marker^21,22^. A significant increase of ATF-3 was detected by qPCR at 14 weeks in untreated *Pla2g6*^−/−^ mice compared to age-matched wild-type controls. However, ATF-3 expression was decreased with semaglutide treatment resulting in a significant reduction in mRNA levels compared to age-matched untreated *Pla2g6*^−/−^ mice (Fig. 2B).

The inhibition of the enzyme glycogen synthase kinase 3 beta (GSK3β) has been found to promote neuroprotection^23,24^. Inversely, cAMP response element-binding protein (CREB) mediates the expression of genes closely associated with neuronal survival, neural differentiation, and neurite outgrowth^25,26^. Therefore, we examined the levels of GSK3β and CREB mRNA using qPCR in the *Pla2g6*^−/−^ model and investigated potential modulation of the expression profile conferred by semaglutide administration, given the improvements in survival and locomotor function observed in Fig. 1 and Fig. S1. The levels of GSK3β present in the brains of end-stage untreated *Pla2g6*^−/−^ mice were significantly higher when compared to age-matched wild-type controls (Fig. 2C). However, treatment with semaglutide ameliorated levels of GSK3β to those measured in wild-type controls and showed a significant difference compared to age-matched untreated *Pla2g6*^−/−^ mice. Additionally, we examined protein levels of phosphorylated GSK3β (P-GSK3β), phosphorylated at the Y216 amino acid residue, which is necessary for enzymatic activity (Fig. S4). The levels of P-GSK3β Y216 protein levels were significantly increased in untreated *Pla2g6*^−/−^ mice, which was restored to wild-type level following treatment with semaglutide (Fig. S4). The levels of CREB gene expression in the brain of untreated *Pla2g6*^−/−^ mice at the humane end-point were found to be significantly lower than those in age-matched wild-type controls (Fig. 2D). Treatment of *Pla2g6*^−/−^ mice with semaglutide significantly increased CREB mRNA levels compared to untreated *Pla2g6*^−/−^ mice and restored expression levels to those observed in the wild-type controls.

To evaluate the effect of 0.5 µg/g semaglutide treatment on neurodegeneration, stereological neuronal counts using the optical fractionator method were conducted on brain sections stained with the pan-neuronal marker, NeuN (Fig. 2F). Stereological counts in the somatosensory barrel field cortex (S1BF) revealed a significant reduction in neuronal numbers in the end-stage untreated *Pla2g6*^−/−^ mice, compared to wild-type controls (Fig. 2E). Treatment with semaglutide resulted in amelioration of neuronal loss, with significantly higher numbers of neurons observed in the treated group compared to untreated controls in the cortex (S1BF). However, this was not restored to wild-type neuronal levels, which were statistically significantly higher in comparison to semaglutide treated *Pla2g6*^−/−^ mice. Amelioration of neurodegeneration resulting from semaglutide treatment was also observed within the ventral posterior medial and lateral nuclei of the thalamus (VPM/VPL) of treated mice (Fig. 2E) compared to untreated controls, however not to wild-type levels.

### Apoptotic and necroptotic cell death in the INAD model is reduced by semaglutide treatment

**Figure 3:**
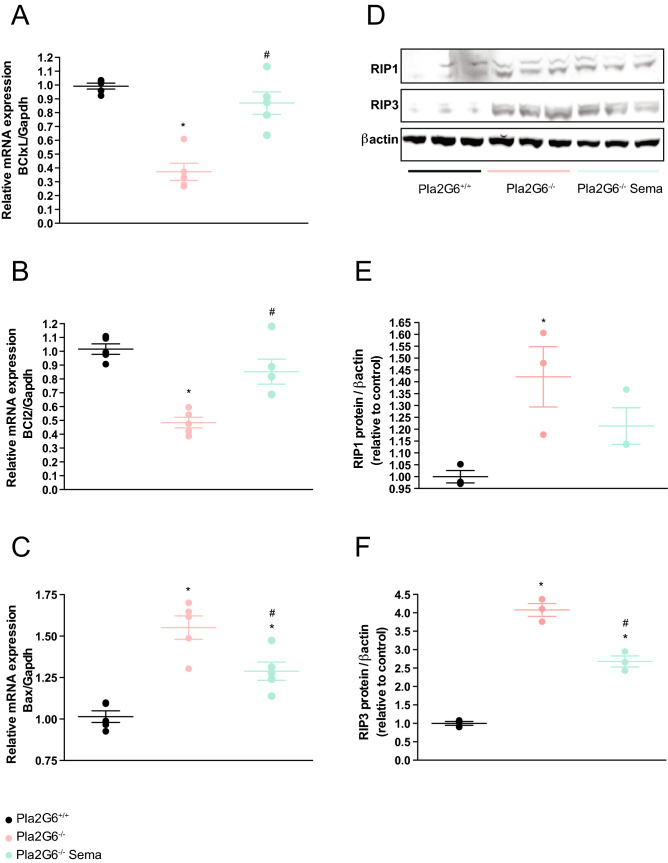
Involvement of apoptosis and necroptosis mechanisms in *Pla2g6*^−/−^ mice treated with semaglutide once weekly. Relative quantification of (**A**) BClxL, (**B**) BCl2 and (**C**) Bax with Gapdh as internal reference by quantitative PCR. RT-qPCR performed from RNA samples prepared from WT, untreated *Pla2g6*^−/−^ and treated *Pla2g6*^−/−^ (*Pla2g6*^−/−^ Sema) brain samples (*n* = 5 per group). (**D**) Western blot analysis and quantification of the expression of (**E**) RIP1 and (**F**) RIP3 in WT, untreated *Pla2g6*^−/−^ semaglutide treated *Pla2g6*^−/−^ brain samples at week 14 (*n* = 3 per group). Protein quantification was normalised with β-actin. All data are presented as mean (SEM); ordinary one-way ANOVA adjusted using Tukey’s multiple comparisons test; *p* values reported in Table S6. * indicates statistically significant difference between the relevant experimental group and WT controls; # indicates statistically significant difference between *Pla2g6*^−/−^ Sema mice and untreated *Pla2g6*^−/−^ controls.

Studies have revealed that common pathophysiological features, including apoptosis and necroptosis, exist in models of rare infantile neurological diseases. An evaluation of cell death was performed using quantification of anti-apoptotic factors such as BCl2 and BClxL, and pro-apoptotic factors such as Bax. This investigation demonstrated involvement of the apoptotic mechanism of cell death in the *Pla2g6-inad* model. Anti-apoptotic factor BClxL is significantly downregulated at end-stage in untreated *Pla2g6*^−/−^ mice compared to wild-type controls (Fig. 3A) (all *p*-values are shown in Supplementary Table S6). Treatment of *Pla2g6*^−/−^ mice with weekly 0.5 µg/g semaglutide increased levels of BClxL to those found in the wild-type controls and statistically significantly increased in comparison to untreated *Pla2g6*^−/−^ mice. Anti-apoptotic factor BCl2 is also downregulated at end-stage in untreated *Pla2g6*^−/−^ mice (Fig. 3B). Treatment of *Pla2g6*^−/−^ mice with semaglutide increased levels of BCl2 to those found in the wild-type controls and significantly increased in comparison to untreated *Pla2g6*^−/−^ mice.

In contrast, the pro-apoptotic factor Bax is upregulated in untreated *Pla2g6*^−/−^ mice compared to age-matched wild-type controls (Fig. 3C). Treatment with semaglutide showed a protective effect in *Pla2g6*^−/−^ mice through significantly reduced Bax expression compared to age-matched untreated *Pla2g6*^−/−^ mice, however this was not completed restored to wild-type levels.

Necroptosis was evaluated as a mechanism of neuronal cell death by western blot (Fig. 3D, Fig. S5). Receptor-interacting protein kinase 1 (RIP1) and RIP3 are key components of the necrosomal complex that regulates necroptotic cell death. We observed increased expression of RIP1 and RIP3 in the brains of end-stage untreated *Pla2g6*^−/−^ mice compared to wild-type controls (Fig. 3E, F). While there was no significant difference in RIP1 levels between treated and untreated *Pla2g6*^−/−^ mice (Fig. 3E), RIP3 was significantly reduced in weekly 0.5 µg/g semaglutide treated mice compared to untreated *Pla2g6*^−/−^ mice, however this was still increased in comparison to age-matched wild-type controls (Fig. 3F).

### Semaglutide treatment ameliorates the neuroinflammatory response in the INAD mouse model

**Figure 4:**
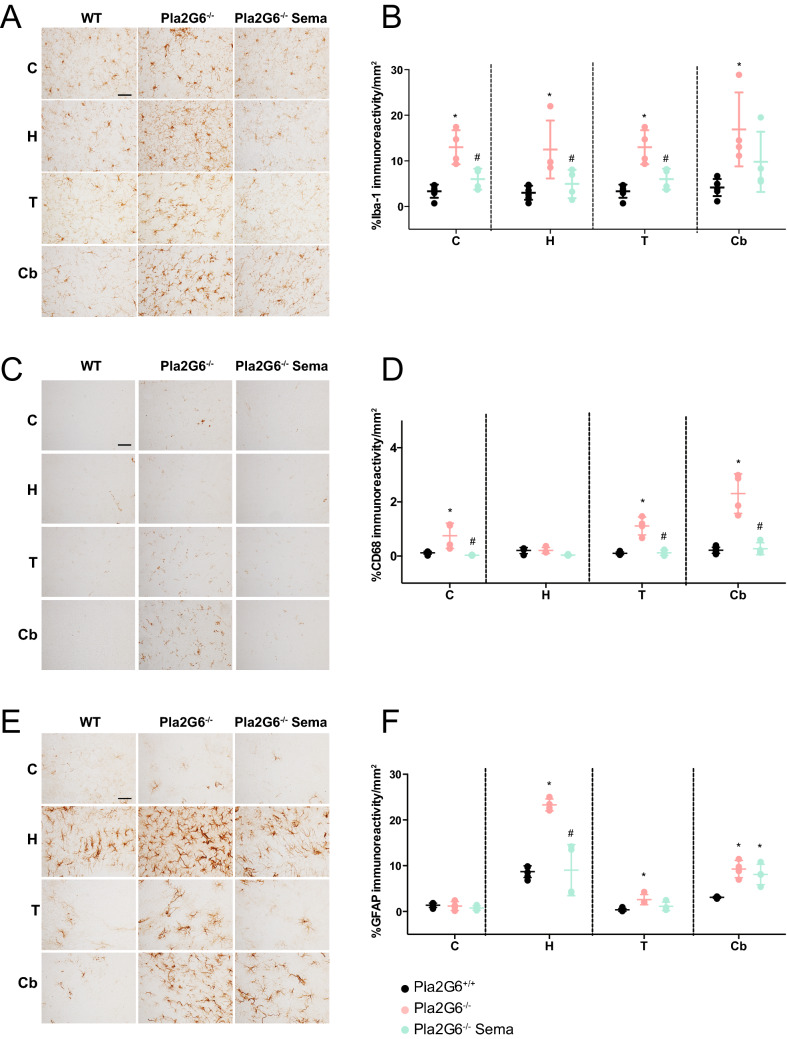
Neuroinflammation in *Pla2g6*^−/−^ mice is reduced by semaglutide treatment. (**A**) Representative micrographs of brain regions (C = cortex, H = hippocampus, T = thalamus, Cb = cerebellum) from Iba-1 stained brain sections and (**B**) neuropathological quantification of Iba-1 positive microglia immunostaining in *Pla2g6*^−/−^ mice treated with 0.5 µg/g semaglutide once weekly (WT *n* = 6, *Pla2g6*^−/−^* n* = 4; *Pla2g6*^−/−^ Sema *n* = 4). Scale bar = 100 µm. (**C**) Representative micrographs of brain regions stained with CD68 and (**D**) quantification of CD68 positive microglia immunostaining in *Pla2g6*^−/−^ mice treated with 0.5 µg/g semaglutide once weekly (WT *n* = 6, *Pla2g6*^−/−^* n* = 4; *Pla2g6*^−/−^ Sema *n* = 4). Scale bar = 100 µm. (**E**) Representative micrographs of brain regions from GFAP stained brain sections and (**F**) quantification of immunoreactivity of GFAP immunostaining in *Pla2g6*^−/−^ mice treated with high-dose semaglutide once weekly (WT *n* = 6, *Pla2g6*^−/−^* n.* = 4; *Pla2g6*^−/−^ Sema *n* = 4). Scale bar = 100 µm. All data are presented as individual animals mean (SEM); ordinary one-way ANOVA adjusted using Tukey’s multiple comparisons test; *p* values reported in Table S7. * indicates statistically significant difference between the relevant experimental group and WT controls; # indicates statistically significant difference between *Pla2g6*^−/−^ Sema mice and untreated *Pla2g6*^−/−^ controls.

Using antibodies against microglia/macrophage-specific calcium-binding protein (Iba-1) and a macrophage-specific marker (CD68), we performed immunohistochemical staining. Both Iba-1 and CD68 are upregulated during microglial activation. This experiment was performed to examine the microglia-mediated inflammatory response in the brains of end-stage (14-week-old) untreated *Pla2g6*^−/−^ mice and the ability of high-dose semaglutide to prevent neuroinflammation. Light microscopy (Fig. 4A, C) and quantitative threshold image analysis (Fig. 4B, D) revealed a significant inflammatory response in specific brain regions in untreated *Pla2g6*^−/−^ mice compared to age-matched wild-type controls (all p-values are shown in Supplementary Table S7). These included the cortex, hippocampus, thalamus, and cerebellum. Microglial activation (Iba-1) was significantly elevated in the untreated *Pla2g6*^−/−^ model compared to wild-type controls (Fig. 4B) in all areas of the brain examined. Microglial activation was significantly reduced by weekly 0.5 µg/g semaglutide treatment in *Pla2g6*^−/−^ mice compared to the untreated controls. This effect was observed throughout all assessed brain regions except in the cerebellum of treated *Pla2g6*^−/−^ mice. Immunohistochemistry using antibodies against CD68 also revealed that untreated *Pla2g6*^−/−^ mice showed an inflammatory response compared to age-matched wild-type controls in all brain regions except in the hippocampus (Fig. 4C, D). Semaglutide treatment significantly reduced the CD68 immunoreactivity in all the affected areas compared to untreated *Pla2g6*^−/−^ mice.

The astrocyte-mediated inflammatory response in the brain was examined by immunohistochemistry using antibodies against glial fibrillary acid protein (GFAP) (Fig. 4E, F). In the untreated *Pla2g6*^−/−^ mice, significant astrogliosis was present in all areas of the brain examined compared to the WT group, with the exception of the cortex (Fig. 4F). Astrogliosis was significantly ameliorated following semaglutide treatment compared to the untreated *Pla2g6*^−/−^ controls. Regional analysis showed semaglutide treatment reduced astrogliosis in the hippocampus brain region compared to the untreated controls, while no statistically significant difference was detected in the thalamus and cerebellum.

### Semaglutide induced neurological improvements partially alters underlying histological pathology

**Figure 5:**
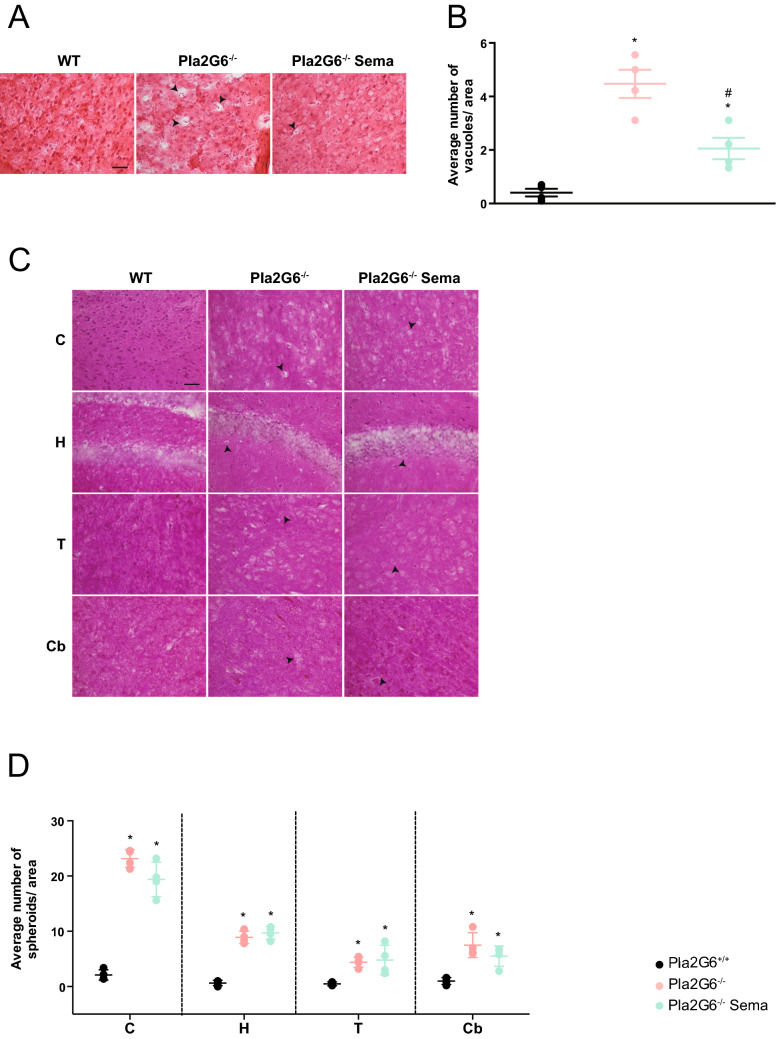
Assessment of INAD disease specific markers: vacuoles and spheroids quantification in *Pla2g6*^−/−^* mice*. (**A**) Representative images and (**B**) vacuole quantification in *Pla2g6*^−/−^ brains using H&E staining (WT *n* = 4, *Pla2g6*^−/−^* n* = 4; *Pla2g6*^−/−^ Sema *n* = 4). Scale bar = 100 µm. (**C**) Representative images of different brain regions stained with periodic acid-Shiff (PAS) staining (C = cortex H = hippocampus T = thalamus Cb = cerebellum). Scale bar = 100 µm. (**D**) Spheroids quantification in *Pla2g6*^−/−^ brains and controls (WT *n* = 4, *Pla2g6*^−/−^* n* = 4; *Pla2g6*^−/−^ Sema *n* = 4). All data are presented as individual animals mean (SEM); ordinary one-way ANOVA adjusted using Tukey’s multiple comparisons test; *p* values reported in Table S8. * indicates statistically significant difference between the relevant experimental group and WT controls; # indicates statistically significant difference between *Pla2g6*^−/−^ Sema mice and untreated *Pla2g6*^−/−^ controls.

H&E staining of brain sections from untreated 14-week-old end-stage *Pla2g6*^−/−^ mice revealed the presence of vacuoles, most noticeably in the cortex (Fig. 5A. Additional images: Fig. S2). Furthermore, PAS staining (Fig. 5C) revealed axonal spheroids bodies that are a characteristic of INAD and have previously been reported in this model^27^. Quantification of vacuoles (Fig. 5B) and spheroids (Fig. 5D) in the brains of untreated mice revealed a significant increase in vacuoles compared to age-matched wild-type controls in the cortex (all p-values are shown in Supplementary Table S8). High-dose semaglutide treatment significantly reduced the number of vacuoles compared to untreated *Pla2g6*^−/−^ mice in the cortex (Fig. 5B), but had no effect on reducing the number of spheroids bodies compared to untreated *Pla2g6*^−/−^ mice in all brain regions (Fig. 5D).

### Repeat high doses of semaglutide does not trigger an inflammatory response in wild-type mice

The effective dose of semaglutide used in this study was 0.5 µg/g. This was administered every week over a 12-week period, from 3 weeks of age. We conducted an examination in wild-type C57BL/6 mice to assess potential inflammatory effects associated with this treatment regimen.

**Figure 6:**
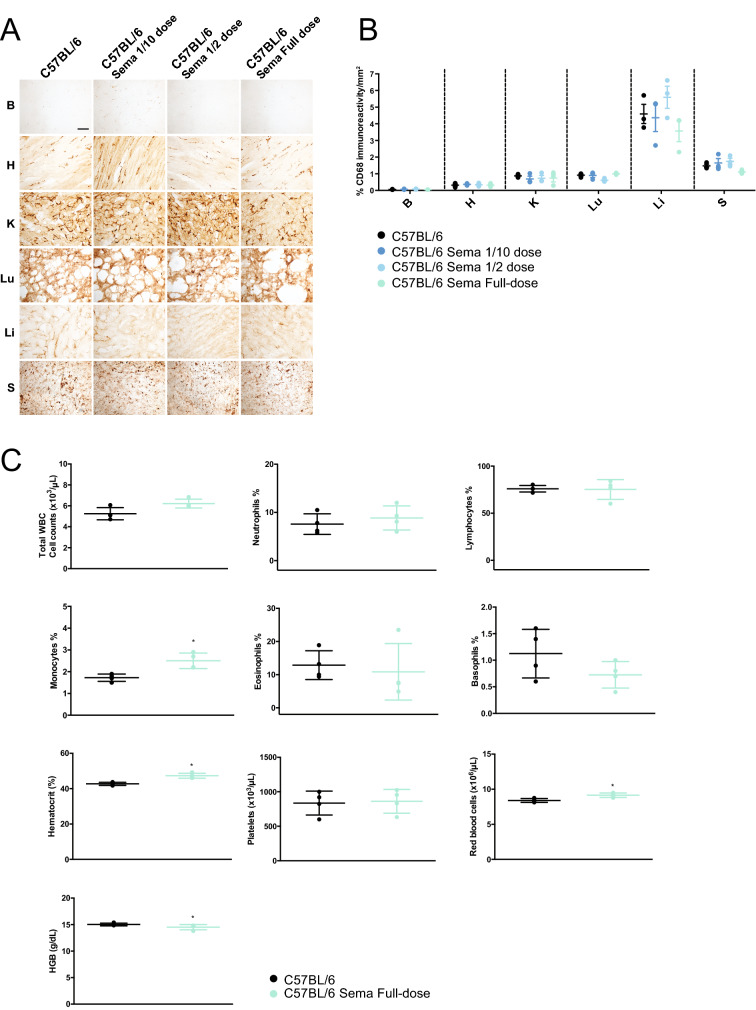
Histopathological organ assessment for macrophage mediated inflammatory response and blood analysis following weekly semaglutide administration. (**A**) Representative images and (**B**) quantitative immunoreactivity threshold measurements of microglial and macrophage marker CD68 in the brain, heart, spleen, liver, lung, pancreas and kidney for the different groups (*n* = 4 per group) in C57BL/6 mice following semaglutide treatment (ip weekly—3 different doses : 1/10 = 0.05 µg/g, half dose = 0.25 µg/g, full-dose = 0.5 µg/g), B = brain, H = heart, K = kidney, Lu = lung, Li = liver, S = spleen. Scale bar = 100 µm. All data are presented as individual animals mean (SEM); ordinary one-way ANOVA adjusted using Dunnett’s multiple comparisons test. (**C**) Total white blood cell (WBC), neutrophils, lymphocytes, monocytes, eosinophils and basophils counts, haematocrit (HCT), platelets, red blood cells (RBC), haemoglobin at the end of the treatment regimen in *Pla2g6*^+*/*+^ mice. All data are presented as individual animals mean (SEM); Mann–Whitney U test; *p* values reported in Table S9. * indicates statistically significant difference between the relevant experimental group and WT controls; # indicates statistically significant difference between *Pla2g6*^−/−^ Sema mice and untreated *Pla2g6*^−/−^ controls.

After completion of whole, half and one tenth dosing regimen of semaglutide, macrophage activation in the brain, heart, spleen, liver, lung, pancreas, and kidney were assessed by immunohistochemistry using an antibody against the macrophage marker CD68 (Fig. 6A). No obvious increase in CD68 staining was observed in any of the experimental cohorts receiving different doses of semaglutide. Furthermore, quantitative threshold image analysis of stained sections (Fig. 6B) did not show any significant macrophage-mediated inflammatory response in any of the organs examined from the semaglutide-administered mice compared untreated wild-type controls (all *p*-values are shown in Supplementary Table S9).

As GLP-1R agonists have been shown to modulate peripheral immune cells^28,29^, blood tests were conducted after completion of the full dose 0.5 µg/g regimen in C57BL/6 mice. Counts of various blood cell populations and biochemistry were performed, including: total white blood cells, neutrophils, lymphocytes, eosinophils, basophils, haematocrit, platelets, red blood cells, and haemoglobin (Fig. 6C). In mice treated with 0.5 µg/g semaglutide regimen, the measured parameters were comparable to wild-type unadministered mice, and no significant differences were observed, with exception of the monocyte count, haematocrit, red blood cells and haemoglobin. These blood counts showed a minor yet significant increase compared to unadministered controls. The increased monocyte count does not exceed 3% and is still within acceptable normal parameters of less than 5% of the total WBC count in mice^30^. Similarly, haematocrit, red blood cell count and haemoglobin levels were slightly increased in injected mice; though, these values are with in the normal haematological range (HCT: 40–55%; RBC: 7.6–11.1 10^6^/µL; HGB: 12–18 g /dL. Source: Central Diagnostic Service, Queen’s Vet School Hospital, University of Cambridge, Cambridge UK).

## Discussion

The importance of GLP-1R agonist as therapies for neurological disorders has recently intensified following positive outcomes in a clinical trial for Parkinson’s Disease patients using exendin-4 (exenatide®)^11^. Our study is the first preclinical assessment of a clinically used GLP-1R agonist for monogenic infantile neurometabolic diseases. Like many of these disorders, INAD is a lethal condition and there is no effective treatment currently available. Here, we demonstrate that 0.5 µg/g semaglutide treatment administered once a week to a mouse model of INAD is neuroprotective, ameliorates neuroinflammation and reduces cell death pathways, resulting in significant improvements to locomotor function and lifespan. Furthermore, this is the first report of a treatment providing a significant extension in lifespan in the *Pla2g6-inad* mouse model. This therapeutic efficacy is achieved without affecting the underlying genetic defect and hallmark spheroid formation. To establish proof-of-concept, the choice of administering the high dose of 0.5 µg/g and via intraperitoneal administration is based on a previous study of neonatal brain injury showing neuroprotective effect of a GLP-1R agonist^9^. Although, semaglutide is usually administered to T2DM patients via subcutaneous injections, or more recently by oral administration (Rybelsus), mouse models of acute brain injury or aggressive neurodegenerative diseases such as INAD may require more rapid entry of semaglutide into the brain which the intraperitoneal route of administration would provide. Follow-up studies examining the effect of subcutaneous administrations are required to assess if this is as effective as the intraperitoneal route and how pharmacokinetics and dynamics differ. We chose to test three doses of semaglutide that were administered weekly: 0.5 µg/g, 0.25 µg/g and 0.15 µg/g. 0.15 µg/g would be the equivalent dose given to adult diabetic patients via subcutaneous administration. Interestingly, only the high dose 0.5 µg/g resulted in a significant increase in lifespan of the INAD mouse model. Although this dose is more than three times higher than the dose given to adult diabetics via subcutaneous administration, it reflects what we have previously shown in a mouse model of hypoxic-ischemic encephalopathy, where a higher dose of 0.5 µg/g is required for therapeutic efficacy in the brain^9^. Further experiments, such as measurement of the levels of semaglutide in blood plasma from injected mice, would provide a better understanding of dosing and the differences between subcutaneous and intraperitoneal administrations.

Neuroprotection was demonstrated by stereological counts of neurons in the brains of semaglutide treated *Pla2g6*^−/−^ mice, indicating an amelioration of neurodegeneration. The formation of vacuoles characteristic of INAD, which arise from the degeneration of axons or synaptic terminals in the cortex^31^, was also reduced following semaglutide treatment. The partial preservation of neurons resulting from the neuroprotective effect of semaglutide represents a potential reason for the reduction in vacuole formation in the brains of the *Pla2g6-inad* model. The reduction of neuronal damage in this model is further demonstrated by the modulation of ATF-3 expression. ATF-3 is one of the most commonly up-regulated transcription factors responding to various types of nerve injuries^22^. In this study, ATF-3 was elevated in untreated *Pla2g6*^−/−^ mice and was normalised to wild-type levels following GLP-1R agonist treatment.

However, the precise mechanisms underlying the neuroprotective properties of GLP-1R agonists are still not fully understood. Several studies have demonstrated neuroprotective action of activated GLP-1R via the PI3K/Akt signalling pathway^32–34^. This promotes key downstream cell survival effectors that can enhance mitochondrial function and reverse neuronal damage, apoptosis and neuroinflammation^35^. Our findings offer further insight into the neuroprotective mechanisms and suggest that a number of pathways are involved. Unexpectedly, although the expression of the *GLP-1R* gene in the untreated *Pla2g6*^−/−^ mice did not differ from WT controls, treatment with semaglutide resulted in up-regulation of *GLP-1R* expression suggesting a feed-back loop upon activation of the receptor. Following activation of the GLP-1 receptor and PI3K/Akt pathway, the neuroprotective effect can be explained by the stimulation of adenylyl cyclase signalling, leading to an increase in the levels of cAMP that activates cAMP response element-binding protein CREB^36^. Modulation of CREB has already been reported in various models of neurodegeneration^25,26^ and in our study, the level of CREB mRNA was reduced in untreated *Pla2g6*^−/−^ mice and normalised in the high-dose semaglutide-treated group. Based on the activation of CREB by the GLP-1R agonist semaglutide, we subsequently investigated changes in the levels of the BCl-2 family, which are known to be involved in the apoptosis pathway and regulated by CREB^37^. It has previously been suggested that phospholipase A2 plays a role in apoptosis and cell death programming^38^. Our study demonstrates the presence of apoptosis through modulation of BCl-2 family members, which was largely reversed by semaglutide treatment. Indeed, mutations in the *Drosophila* homologue of the *PLA2G6* gene, *iPLA-*VIA, results in mitochondrial abnormalities such as respiratory chain dysfunction, abnormal mitochondrial morphology and subsequent reduced ATP synthesis^39^. Furthermore, a knock-out mouse model of INAD shows abnormal mitochondria with degenerative inner membranes^40^. A number of publications report beneficial effects of GLP-1R agonists on enhancing mitochondrial biogenesis^41^ and restoring membrane potential and function^42^. This evidence raises the potential of GLP-1R agonists for treating both rare and more common neurological diseases with a strong mitochondrial component e.g. PRKN- or PINK1-associated Parkinson’s Disease. Our findings also confirm activation of the necroptotic pathway with upregulation of RIP1 and RIP3 proteins for the first time in a *Pla2g6*^−/−^ mouse model. This upregulation was reduced following treatment with the GLP-1R agonist.

Alternatively, the neuroprotection resulting from semaglutide treatment may be induced via the modulation of glycogen synthase kinase-3β (GSK3β), a multifunctional serine/threonine kinase which is also regulated through the PI3K/Akt pathway, where activation of the pathway inhibits its activity^43^. GSK3β overexpression leads to an increase in disease-induced neurodegeneration^44^ and plays a strong proinflammatory role in many CNS diseases^45^. Our data confirms the overexpression of GSK3β with significantly increased levels observed in untreated *Pla2g6*^−/−^ mice. Moreover, treatment with the GLP-1R agonist affects the transcriptional regulation of GSK3β resulting in significant reduction and normalisation to wild-type levels. The regulation of GSK3β is complex, with activation potentially occurring via phosphorylation on Try216/279 and inactivation via phosphorylation on Ser9/21^44^. More studies are required to investigate regulation at the protein level, and effects on the phosphorylation status of GSK3β following induction by GLP-1R agonists to further elucidate the mechanism of action.

Semaglutide treatment does not address the underlying genetic metabolic defect, yet it reduced the extent of neuroinflammation and cell death, demonstrating its role as a neuroprotective drug. Combined with the fact that semaglutide already has approval for use in Type 2 diabetes mellitus and a good safety record, there is potential for semaglutide and other GLP-1R agonists to be repurposed as neuroprotective therapeutic agents. This is relevant not only for the disease evaluated in this study, but more widely for other paediatric neurometabolic diseases for which there is no available therapy. There are currently no effective treatments for INAD^46^ and semaglutide represents a new potential therapeutic agent that could decelerate disease progression and potentially improve the patient’s quality of life. This preclinical and proof-of-concept study in a model of a rare infantile neurometabolic disorder is an essential first step to potentially advance the use of semaglutide for clinical benefit. The fact that the drug induced neuroprotection in multiple brain regions suggests that it could reduce neurodegeneration independent of the mechanism and site of individual disease pathology. This therefore promotes the potential use of the drug in a broad spectrum of neurodegenerative disorders, many of which may not yet have alternative treatments available and irreversibly affect children. While semaglutide is not approved for use in children yet, the treatment is currently being tested in a phase 3 clinical trial with the aim to investigate efficacy and safety of semaglutide in children and teenagers with type 2 diabetes mellitus (ClinicalTrials.gov Identifier: NCT04596631). In addition, in 2019 the FDA approved the use of liraglutide for the treatment of Type 2 diabetes mellitus in children^47^. Semaglutide could be more effective than other GLP-1R agonists in its neuroprotective action due to its longer half-life of 7 days^48^, and could elicit fewer immune responses in patients due to its 94% homology to human GLP-1 protein^18^. Preclinical studies using semaglutide for treating different types of neurological diseases such as Parkinson’s disease^19,20^ and stroke^10^ also show promising results and for the first time, our study shows beneficial results in a paediatric neurometabolic disorder. All these studies support the continued investigation of semaglutide for future clinical translation as a treatment for a broad spectrum of neurodegenerative disorders.

## Materials and methods

### Study design

Our objective was to characterise the effect of semaglutide in the *Pla2g6-inad* mouse model. The control and treatment groups and the number of biological replicates (sample sizes) for each experiment are specified in the figure legends. For the in vivo studies, animals were randomly allocated to the control and treatment groups and housed together to minimise environmental differences and experimental bias. Double blinding was used with one person assigned to perform the behavioural assessments and second completed the analysis, with disease symptoms becoming evident towards the end of the protocol.

### Animals

All experiments were performed on *Pla2g6*^−/−^ mice from 3 weeks to at least 14 weeks of age. Predetermined parameters were set to establish the humane endpoints. A loss of 10% body weight was established as a humane endpoint. Additional euthanasia criteria included changes in appetite, unrelieved pain/distress, organ system dysfunction or failure and clinical signs which indicate that the animal has entered a moribund state.

All mice were housed in grouped cages in a temperature-controlled environment with food and water given ad libitum. All animal experiments were approved by the Ethics Committee of the University College London and carried out by licensed personnel in concordance with the UK Home Office Guidelines [Animals (Scientific Procedures) Act, 1986] and in compliance with the ARRIVE guidelines.

The *Pla2g6-inad* mouse model was generated using the n-ethyl-nitrosourea (ENU) chemical mutagen. Sequencing of affected mice revealed a single mutation in the open reading frame sequence of the *Pla2g6* gene. The mutation was identified as a G to A transition at base 1117, resulting in a non-synonymous amino acid exchange from glycine to arginine at position 373^27^. This mutation is a point mutation (G373R) localized in the Ankyrin repeat of the *Pla2g6* gene. Of critical importance, it is the same mutation identified in patients with INAD^1^. Behavioural studies in this mouse model have shown that motor dysfunction begins by the age of 7 to 8 weeks, where all homozygous affected mice start to display abnormal movement and muscular deficits. Homozygous mutants become emaciated and die before 18 weeks of age. Histological analyses of the muscles reveal mass atrophy in the hind limb muscles, suggesting neural degeneration and peripheral nervous system dysfunction. Heterozygote littermates show no evidence of gross abnormality compared with WT mice suggesting that the INAD symptoms are inherited as an autosomal recessive trait in the same way as human INAD patients. Widespread formation of spheroids containing tubulovesicular membranes can be observed in these animals^27,49^.

Mice were divided at random into different groups: knock-in saline and knock-in semaglutide groups, and compared to wild-type controls. Semaglutide solution was prepared in 0.9% (w/v) NaCl solution. The semaglutide treatment group was administered with an intraperitoneal injection of semaglutide (Bachem, 4091661) every week starting at 3 weeks of age, and then for the duration of the animal’s life (until the humane end-point was reached) at three different doses: 0.5 µg/g, 0.25 µg/g and 0.15 µg/g. Experimental mice were randomly allocated from litters to each experimental cohort and the sex distribution was as follows: wildtype (WT) *Pla2g6*^+*/*+^ (3 males; 3 females), untreated *Pla2g6*^−/−^ (4 males; 2 females), *Pla2g6*^−/−^ treated with weekly 0.5 µg/g semaglutide (3 males; 3 females), *Pla2g6*^−/−^ treated with weekly 0.25 µg/g semaglutide (1 male, 5 females) and *Pla2g6*^−/−^ treated with weekly 0.15 µg/g semaglutide (2 males; 4 females). The group sizes were determined by a power calculation (power 0.8) using PS Power and Sample size software^50^. The calculation was based upon differences between wildtype and mutant INAD mice in a previous report^27^.

### Genotyping

The ear punch method was used for genotyping the animals. DNA was extracted by adding 25 Mm NaOH, containing 0.2 mM EDTA N2 and 40 mM Tris–HCl, to the sample tissue. Samples were vortexed and heated to 95ºC for 20 min. Once the DNA was extracted, allele specific primer PCR was used to identify the genotype of each animal. The primers were designed to detect either a wild-type (WT) or a mutant allele (KO), depending on the number of base pairs of the PCR products (see Table S3).

The genotyping protocol was developed based on the ASP (Allele specific primer)–PCR method developed by Pr. Ken-Ichiro Seino’s group (St Marianna University, Japan). The forward primer contains a mismatch at the third nucleotide from the 3’ end to enhance PCR specificity. The wildtype and mutant alleles are designed to result in respectively a 346 bp and 617 bp PCR products.

### Behavioural analysis

The animals were pre-trained for all tasks and tested at approximately the same time each week. Animals were weighed at weekly intervals. Behavioural testing was commenced post-weaning at week 5 and conducted weekly, until week 14 for all three cohorts (untreated *Pla2g6*^−/−^*,* high-dose semaglutide (0.5 µg/g) treated *Pla2g6*^−/−^ and wild-type mice).

#### Rotarod

Motor coordination and balance were assessed using an accelerating rotarod (Panlab, Harvard apparatus, Cambridge, UK). Mice were first habituated to the rotating rod at a constant speed of 4 rpm for 300 s (5 min), during which time latency to fall was not recorded. The habituation trial was only performed on the first day. Mice were subsequently exposed to a rotating rod starting at 4 rpm and linearly accelerated to 40 rpm over a 5 min period. Three trials were realized per day with a maximum time of 5 min.

#### Vertical pole test

The vertical pole test is widely used to monitor motor impairments in animals. Mice placed on a vertical pole (1 cm diameter 60 cm high), turn downwards and descend the pole. Mice were habituated to the task in 2 trials per day for 2 days. On test day (third day), the total descent time was measured was over 3 trials per mouse. If the animal failed the test by not actively descending but falling, the maximum of 10 s was recorded for the animal.

#### Open field test

The Open Field activity monitoring system comprehensively assesses locomotor and behavioural activity levels in mice. The apparatus is constructed of clear Plexiglas and measured 72 × 72 cm with 36 cm high walls. Each mouse was placed in a corner of the open field and allowed to familiarise itself with the space for 5 min, before the mouse was recorded freely exploring the apparatus for 5 min. Measures of total distance travelled, duration of immobility and mobility, average speed were obtained with an automated camera-based computer tracking software (AnyMaze, Rathmines, Ireland). Following the 5 min test, mice were returned to their home cages and the open field was cleaned with 70% of Ethanol and permitted to dry between tests. To assess the process of habituation to the novelty of the arena, mice were exposed to the apparatus for 5 min on 2 consecutive days.

### Staining analysis

Mice were sacrificed at the end of the study. The brain and other organs (heart, lung, liver, pancreas, spleen, and kidney) of mice treated with weekly 0.5 µg/g semaglutide were fixed via cardiac perfusion with phosphate-buffered saline (PBS), dissected out and postfixed at 4^∘^C in 4% paraformaldehyde (PFA 4%) for at least 24 h and cryoprotected in 30% sucrose solution overnight. All staining except H&E were performed on 40 μm thick free-floating brain and organ sections. H&E staining was performed on 40 μm-thick sections that were pre-mounted onto slides.

### Haematoxylin and eosin staining

The brain and organ sections were stained with H&E for the histological studies.

### PAS staining

PAS staining was performed to visualise the glycosylated components of tissue with the PAS staining kit 101,646 from Merck. The mounted brain sections were first immersed in periodic acid solution for 5 min at room temperature, and then rinsed in distilled water. The slides were then immersed in Schiff’s reagent for 15 min at room temperature and rinsed in running tap water for 5 min. The sections were then counterstained with haematoxylin solution for 90 s and rinsed again in tap water. The sections were finally immersed with IMS and xylene before fixation with DPX mounting medium with coverslips.

### Immunohistochemistry

After pre-treatment in 1% H_2_O_2_ and 15% normal serum (Vector), sections were stained overnight using anti-CD68/NeuN/GFAP/Iba-1 antibodies (reference in Table S2) with 10% normal serum in TBS-T. Sections were then incubated with biotinylated secondary antibodies (dilution 1:1000, Vector Laboratories) and subsequently processed using avidin-biotinylated peroxidase (Vectastain ABC kit, dilution 1:1000, Vector Laboratories). Quantification was performed with Image Pro Premier software.

### Western blot

The expression of proteins extracted from brain samples of mice was analysed by Western blot. The protein samples were separated on 4–12% Bis–Tris protein gels (NuPage, Thermofisher) and transferred onto polyvinylidene fluoride membranes (Millipore, USA). The membranes were incubated with RIP1, RIP3 and P-GSK3β Y216 antibodies (Cell Signalling Technology, USA) at 4ºC overnight. Immunoreactive bands were visualized by increased chemiluminescence using corresponding horseradish peroxidase-conjugated IgG secondary antibodies. The images were captured by the gel imager (UVP Chemstudio) and quantified using VisionWorks and ImageJ softwares. Images of the full-length original blots are available in the Supplementary Figures S4 and S5.

### qPCR

To assess *GLP-1R* gene expression in *Pla2g6*^−/−^ mice, with or without GLP1-R agonist treatment, brains were extracted, and total RNA was isolated using RNeasy Mini kit (Qiagen). First-strand cDNA was then generated using the High-capacity cDNA Reverse Transcription kit (Applied Biosystems). Quantitative RT-PCR was carried out using a Step One Plus Real-Time PCR system (Applied Biosystems) with the SsoAdvanced Universal SYBR Green PCR Core Reagents Supermix (Biorad). *GSK3β* and *CREB* gene expression were also measured to study the neuroprotective properties of the treatment. ATF-3 is a neuronal damage marker and was also measured by qPCR. Apoptosis status was assessed by measuring BCl2, BClxL and Bax expression levels. All primers were designed or found in literature (see Table S1). Data were calibrated to Gapdh and the relative quantitation of gene expression was performed using the comparative Ct method.

#### Stereology

Stereology analysis was carried out on 40 μm brain sections stained for the pan-neuronal marker NeuN using StereoInvestigator software (MBF Bioscience, USA) on a Nikon Optiphot microscope (Nikon) attached to a Q-Imaging Model 01-MBF-2000R-CLR-12 camera (MBF Bioscience). Estimation of neuronal numbers within the cortex (S1BF) and thalamus (VPM/VPL) was conducted using the optical fractionator probe. Using a 100 × objective, NeuN positive cells were identified and counted. A border was traced around the region of interest, a grid was superimposed, and neurons were counted within a series of 50 × 50 μm dissector frames, which were arranged according to the sampling grid size. Every sixth section containing the region of interest was analysed and grid sizes were 225 × 225 μm^2^ for the cortex and 175 × 175 μm^2^ for the thalamus. A coefficient of error (CE) between 0.05 and 0.1 was obtained for all counts indicating sufficient sampling efficiency^51^.

#### Blood analysis

To assess any haematological effects of semaglutide treatment, mice received semaglutide injections from week 3 to week 14 via intraperitoneal administration (0.5 µg/g per dose, weekly). Naïve mice acted as controls. 7 days after the last injection, blood samples taken via cardiac puncture and collected in an EDTA-coated tube. The analysis was performed by MRC Harwell Clinical Pathology laboratory (Mary Lyon Centre, UK). Parameters measured included: total white blood cell count, neutrophils, lymphocytes, monocytes, eosinophils and basophils count, haematocrit, platelets, red blood cells, haemoglobin and mean corpuscular volume.

#### Statistical analysis

Data were analysed using the Graphpad Prism software program Version 7.0. Statistical analysis was performed using a two-way or one-way analysis of variance (ANOVA—depending on the analysis) and a Dunnett’s or Tukey’s multiple comparisons test. A Mann–Whitney U test was used to compare blood parameters. The * symbol indicates a significant difference between the control WT group and either untreated *Pla2g6*^−/−^ (WT vs *Pla2g6*^−/−^) or treated *Pla2g6*^−/−^ mice (WT vs *Pla2g6*^−/−^ Semaglutide); the # symbol indicates a significant difference between the treated *Pla2g6*^−/−^ Semaglutide group and the untreated *Pla2g6*^−/−^ group. All *p* values are reported in Tables S4–S10. Data are presented as mean (SEM).

## Supplementary Information

Supplementary Information.

## Data Availability

All data generated or analysed during this study are included in this published article and supplementary information files. Raw data are available from the corresponding author on reasonable request.
