# Supplementary material for: A GLP1 receptor agonist diabetes drug ameliorates neurodegeneration in a mouse model of infantile neurometabolic disease
Source: Sci Rep. 2022 Aug 15;12:13825. doi: 10.1038/s41598-022-17338-1 (PMC9378686; doi:10.1038/s41598-022-17338-1)
Supplement: Supplementary file 1 — Supplementary Information. [file 41598_2022_17338_MOESM1_ESM.pdf]

Supplementary Materials

Fig. S1. Improvement of locomotor symptoms in *Pla2g6*$^{-/-}$ mice with weekly Semaglutide treatment.

Fig. S2. H&E staining of brain (**A**) and visceral organs (**B**) sections.

Fig. S3. Ct Gapdh Values for qPCR.

Fig. S4. P-GSK3β Y216 Western blot and quantification.

Fig. S5. Full length Western blots of RIP, RIP3 and β-actin.

Table S1. Primers sequence table for qPCR.

Table S2. Summary table of antibodies used for the ICH and western blot.

Table S3. Forward and Reverse Primers sequences table for genotyping.

Table S4. *P* values for Fig 1.

Table S5. *P* values for Fig 2.

Table S6. *P* values for Fig 3.

Table S7. *P* values for Fig 4.

Table S8. *P* values for Fig 5.

Table S9. *P* values for Fig 6.

Table S10. *P* values for Fig S1.

Table S11. *P* values for Fig S3.

Fig. S1. Improvement of locomotor symptoms in Pla2g6$^{-/-}$ mice with weekly Semaglutide treatment.

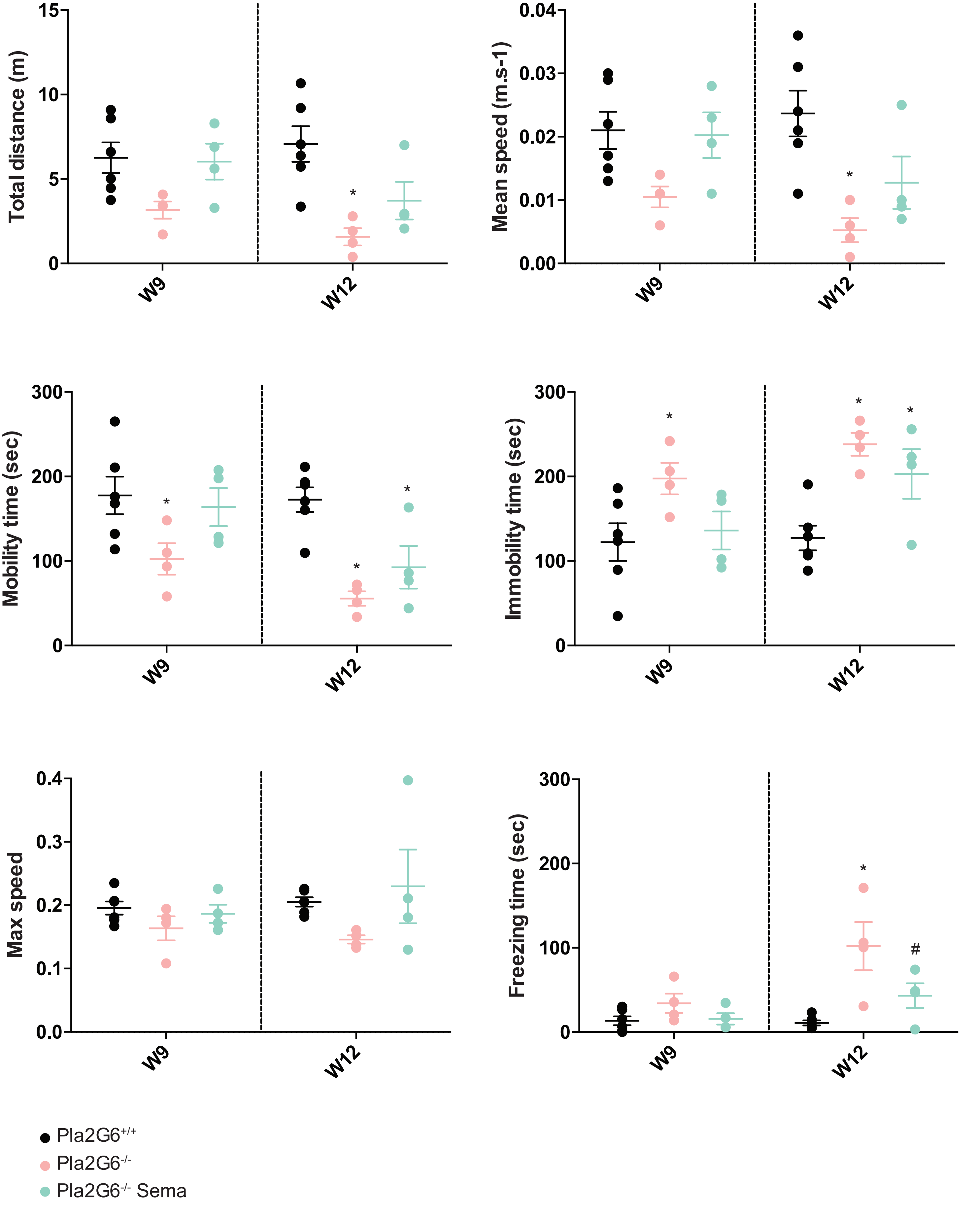

Fig. S2. H&E staining of brain (A) and visceral organs (B) sections.

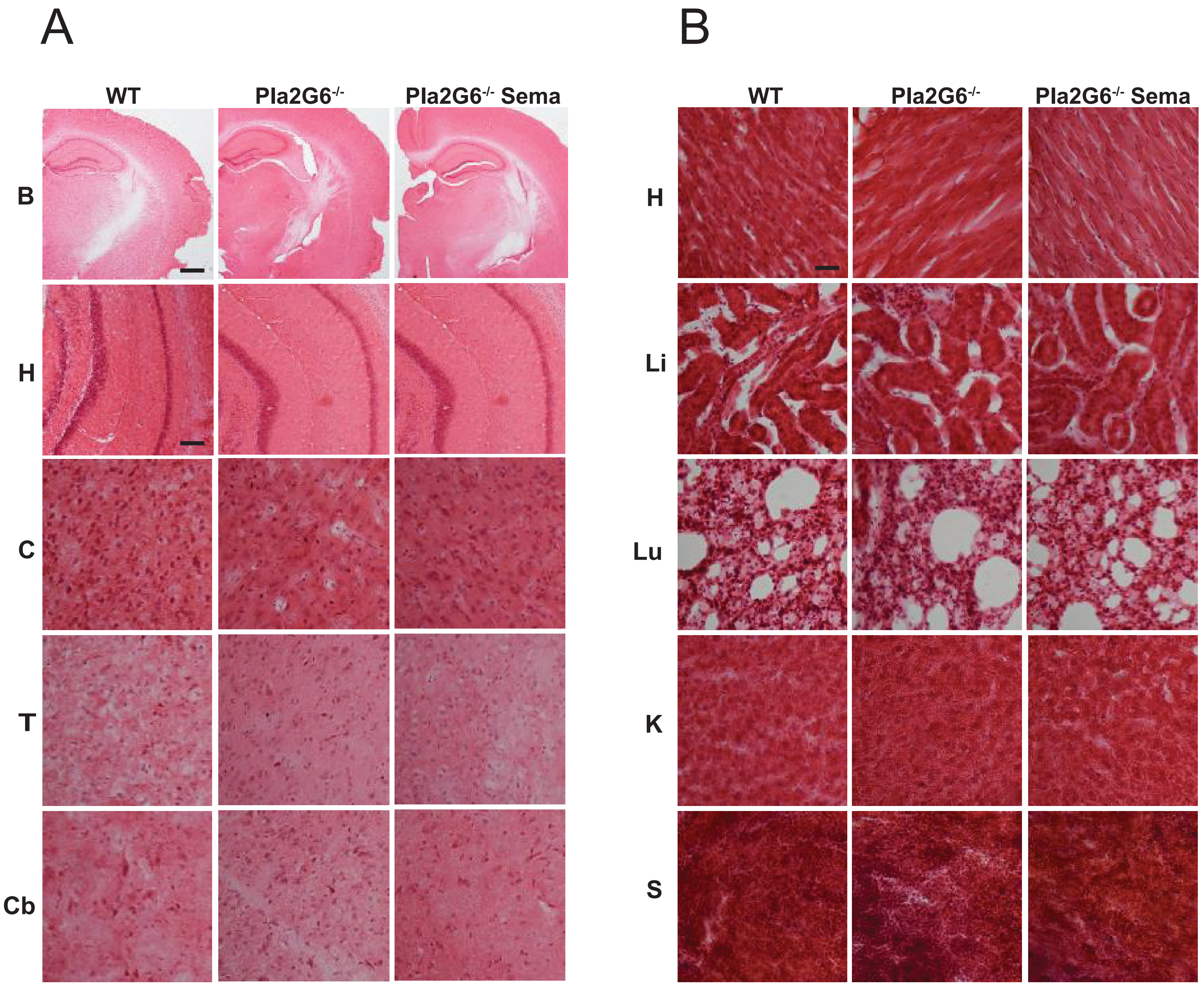

Fig. S3. Ct Gapdh Values for qPCR.

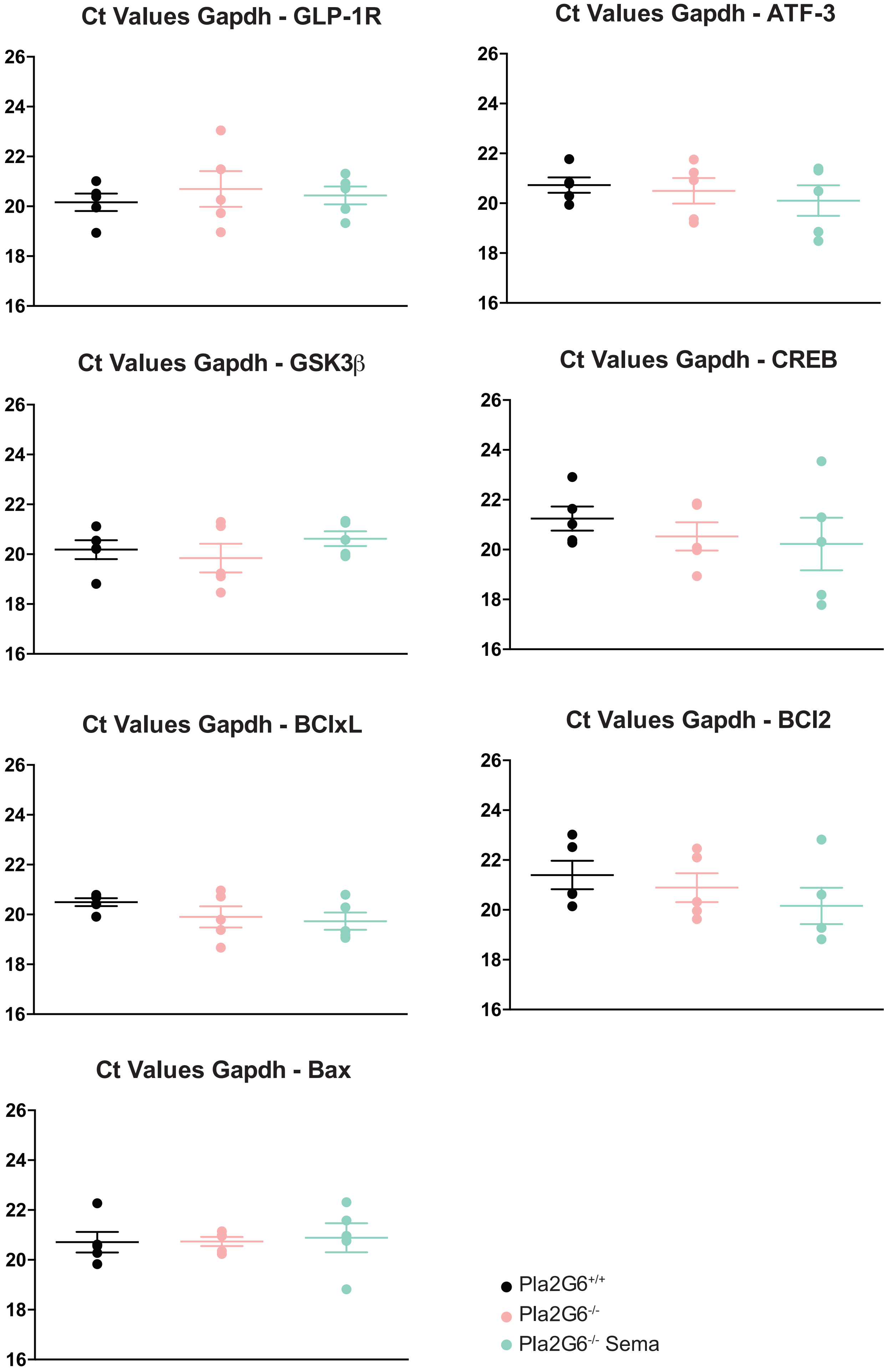

Fig. S4. P-GSK3β Y216 full-length blot.

P-GSK3β Y216 blot

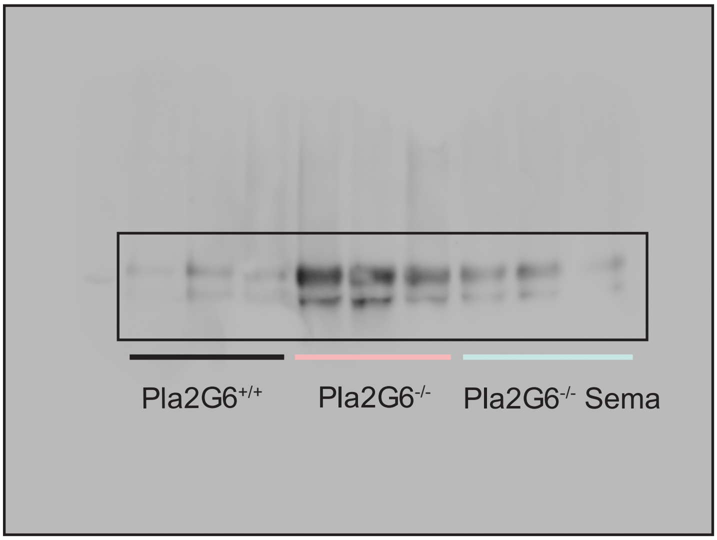

Actin blot

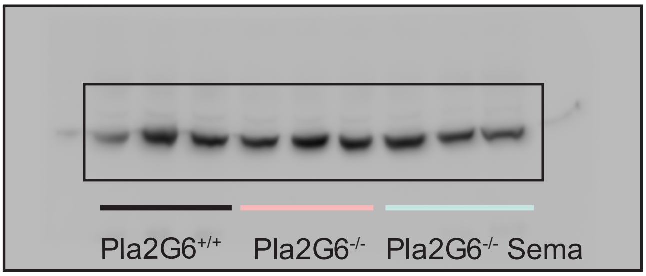

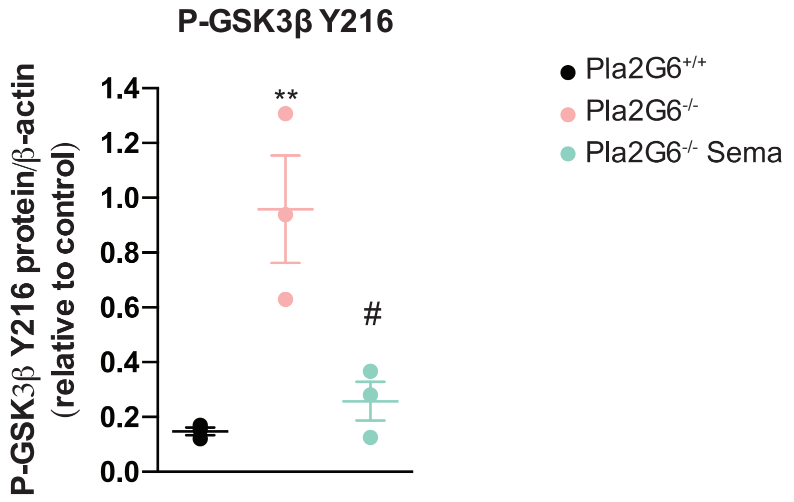

Table S12	
	***P* value**
**Pla2G6$^{+/+}$ vs. Pla2G6$^{-/-}$**	0.0075
**Pla2G6$^{+/+}$ vs. Pla2G6$^{-/-}$ Sema**	0.8011
**Pla2G6$^{-/-}$ vs. Pla2G6$^{-/-}$ Sema**	0.0148

Fig. S5. Full-length blots.

RIP1 blot

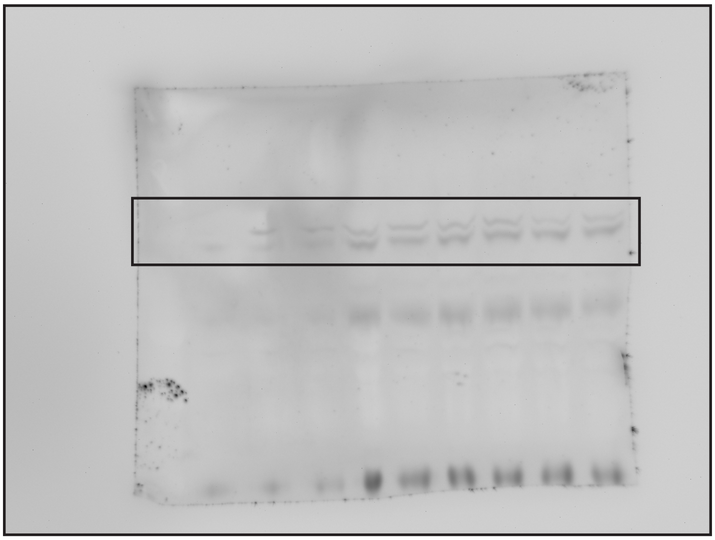

RIP3 blot

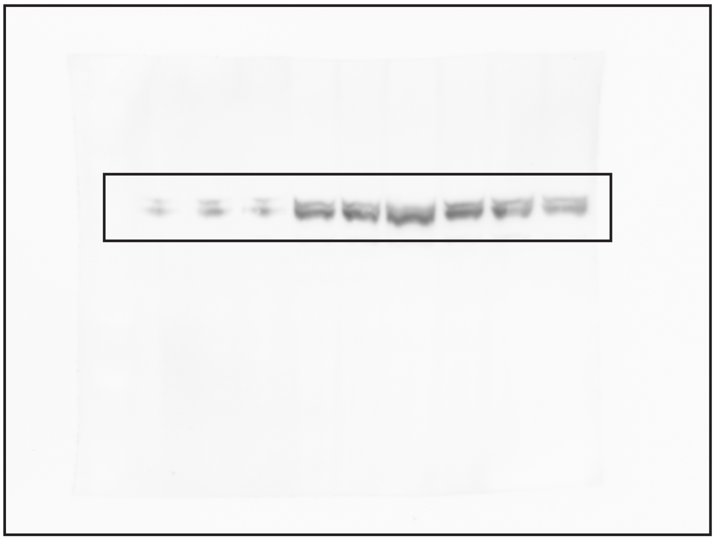

Actin blot

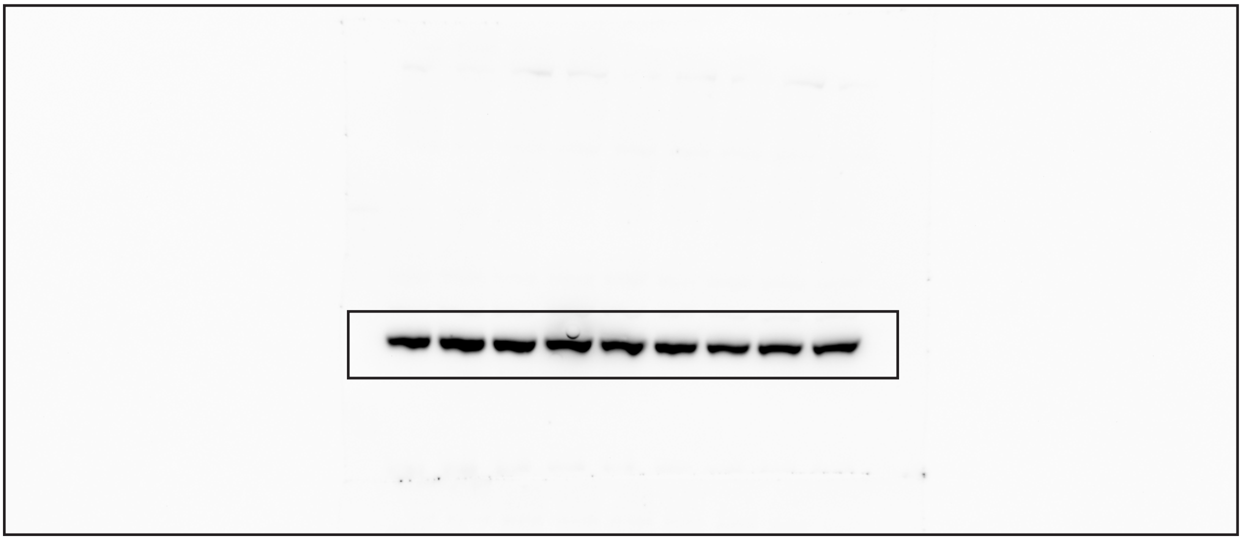

Supplementry Data Tables

Table S1 Primer sequences for qPCR.

Target Gene	Sequence	Reference
Gapdh	F GTTGTCTCCTGCGACTTCA<br>R GGTGGTCCAGGGTTTCTTA	Pick primers
CREB	F CACAGAACCAGTTTCCATCATCCAGT<br>R CATGTTCAGAGGGTTAGGGAGAGCA	Miller, 2012
GSK3β	F TTGGACAAAGGTCTTCCGGC<br>R AAGAGTGCAGGTGTGTCTCG	Li, 2015
GLP1-R	F AGACGGTGCAGAAATGGAGA<br>R TGGCGCTTCCGTGAGG	During, 2003
ATF-3	F TTACCGTCAACAACAGACCC<br>R TCAGCTCAGCATTCACACTC	Edagawa, 2014
BCl2	F GTGGTGGAGGAACTCTTCAG<br>R GTTCCACAAAGGCATCCCAG	Wenyuan, 2016
BClxL	FCGGAGAGCGTTCAGTGATCTA<br>R TTGTCTACGCTTTCCACGCA	Pick primers
Bax	F AGCAAACTGGTGCTCAAGGC<br>R CCACAAAGATGGTCACTGTC	Wenyuan, 2016

Table S2. Summary of primary antibodies.

Antibody	Reference	Dilution	Protocol
CD68	MCA1957	1:2000	ICH
NeuN	MAB377	1:500	ICH
Iba-1	ab5076	1:500	ICH
GFAP	MAB3402	1:2000	ICH
RIP1	ab72139	1:1000	WB
Actin	ab8226	1:5000	WB
P-GSK3β Y216	ab75745	1:1000	WB

Table S3. Forward and Reverse primers for genotyping.

Gene	Sequence	Size
Pla2G6<br>WT	R: CTCTGCTTGTTCTGCTGCTG<br>F: GTCAAAGCCCTCATTGTATTCGG	346 bp
Pla2G6<br>Mutant	R: GGAGGGTCGGGTCCTATTTA<br>F: GTCAAAGCCCTCATTGTATTCAG	617 bp

Table S4. P values for Fig. 1.

	% Survival
Pla2G6$^{-/-}$ vs. Pla2G6$^{-/-}$ Sema 0.5ug/week	<0.001
Pla2G6$^{-/-}$ vs. Pla2G6$^{-/-}$ Sema 0.25ug/week	0.0505
Pla2G6$^{-/-}$ vs. Pla2G6$^{-/-}$ Sema 0.15ug/week	0.5107
Pla2G6$^{-/-}$ Sema 0.25ug/week vs. Pla2G6-/- Sema 0.15ug/week	>0.9999
Pla2G6$^{-/-}$ Sema 0.25ug/week vs. Pla2G6-/- Sema 0.5ug/week	0.7429
Pla2G6$^{-/-}$ Sema 0.15ug/week vs. Pla2G6-/- Sema 0.5ug/week	0.0457

Figure 1D

	Age (weeks)									
	W5	W6	W7	W8	W9	W10	W11	W12	W13	W14
Pla2G6$^{+/+}$ vs. Pla2G6$^{-/-}$	0.7601	0.5981	0.5507	0.448	0.3947	0.0163	0.0003	0.0161	0.0087	0.0058
Pla2G6$^{+/+}$ vs. Pla2G6$^{-/-}$ Sema	0.9996	0.9989	0.9972	0.8484	0.9616	0.9139	0.1149	0.4664	0.3332	0.3469
Pla2G6$^{-/-}$ vs. Pla2G6$^{-/-}$ Sema	0.7818	0.6258	0.6488	0.2473	0.6104	0.0773	0.1475	0.3022	0.314	0.2473

Figure 1E

	Age (weeks)									
	W5	W6	W7	W8	W9	W10	W11	W12	W13	W14
Pla2G6$^{+/+}$ vs. Pla2G6$^{-/-}$	0.4734	> 0,9999	0.9974	0.2104	0.0002	< 0,0001	< 0,0001	< 0,0001	< 0,0001	< 0,0001
Pla2G6$^{+/+}$ vs. Pla2G6$^{-/-}$ Sema	0.944	0.9965	0.9275	0.9495	0.9976	0.3005	0.0998	0.0006	0.0001	< 0,0001
Pla2G6$^{-/-}$ vs. Pla2G6$^{-/-}$ Sema	0.7204	0.9964	0.9154	0.4152	0.0008	< 0,0001	< 0,0001	0.0016	0.0033	0.3467

Table S5. P values for Fig. 2.

	GLP1-R mRNA *P* value	ATF-3 mRNA *P* value	GSK3β mRNA *P* value	CREB mRNA *P* value	S1BF NeuN *P* value	VPM/VPL NeuN *P* value
Pla2G6$^{+/+}$ vs. Pla2G6$^{-/-}$	0.6872	< 0,0001	0.0001	0.0003	< 0,0001	< 0,0001
Pla2G6$^{+/+}$ vs. Pla2G6$^{-/-}$ Sema	0.0003	0.1873	0.9506	0.9617	< 0,0001	< 0,0001
Pla2G6$^{-/-}$ vs. Pla2G6$^{-/-}$ Sema	< 0,0001	0.0005	< 0,0001	0.0002	0.0006	0.0012

Table S6. P values for Fig. 3.

	BClxL mRNA *P* value	Bcl2 mRNA *P* value	Bax mRNA *P* value	RIP1 protein *P* value	RIP3 protein *P* value
Pla2G6$^{+/+}$ vs. Pla2G6$^{-/-}$	< 0,0001	0.0001	< 0,0001	0.0329	< 0,0001
Pla2G6$^{+/+}$ vs. Pla2G6$^{-/-}$ Sema	0.3552	0.1814	0.0115	0.2692	0.0003
Pla2G6$^{-/-}$ vs. Pla2G6$^{-/-}$ Sema	0.0002	0.0028	0.015	0.2867	0.0009

Table S7. P values for Fig. 4.

	Iba-1			
	Cortex *P* value	Hippocampus *P* value	Thalamus *P* value	Cerebellum *P* value
Pla2G6$^{+/+}$ vs. Pla2G6$^{-/-}$	0.0002	0.0072	0.0002	0.0122
Pla2G6$^{+/+}$ vs. Pla2G6$^{-/-}$ Sema	0.2666	0.7235	0.2666	0.3026
Pla2G6$^{-/-}$ vs. Pla2G6$^{-/-}$ Sema	0.0057	0.043	0.0057	0.2176

	CD68			
	Cortex *P* value	Hippocampus *P* value	Thalamus *P* value	Cerebellum *P* value
Pla2G6$^{+/+}$ vs. Pla2G6$^{-/-}$	0.0059	> 0,9999	< 0,0001	< 0,0001
Pla2G6$^{+/+}$ vs. Pla2G6$^{-/-}$ Sema	0.832	0.0648	0.982	0.9761
Pla2G6$^{-/-}$ vs. Pla2G6$^{-/-}$ Sema	0.0043	0.0641	< 0,0001	< 0,0001

	GFAP			
	Cortex *P* value	Hippocampus *P* value	Thalamus *P* value	Cerebellum *P* value
Pla2G6$^{+/+}$ vs. Pla2G6$^{-/-}$	0.8969	< 0,0001	0.0028	0.0015
Pla2G6$^{+/+}$ vs. Pla2G6$^{-/-}$ Sema	0.3454	0.9847	0.3357	0.006
Pla2G6$^{-/-}$ vs. Pla2G6$^{-/-}$ Sema	0.6355	0.0001	0.0539	0.6126

Table S8. P values for Fig 5.

	Vacuoles *P* value
Pla2G6$^{+/+}$ vs. Pla2G6$^{-/-}$	0.0001
Pla2G6$^{+/+}$ vs. Pla2G6$^{-/-}$ Sema	0.0375
Pla2G6$^{-/-}$ vs. Pla2G6$^{-/-}$ Sema	0.0047

	Spheroids			
	Cortex *P* value	Hippocampus *P* value	Thalamus *P* value	Cerebellum *P* value
Pla2G6$^{+/+}$ vs. Pla2G6$^{-/-}$	< 0,0001	< 0,0001	0.014	0.0003
Pla2G6$^{+/+}$ vs. Pla2G6$^{-/-}$ Sema	0.0001	0.0001	0.0025	0.0008
Pla2G6$^{-/-}$ vs. Pla2G6$^{-/-}$ Sema	0.2637	0.2068	0.4889	0.7448

Table S9. P values for Fig. 6.

	CD68					
	Brain *P* value	Heart *P* value	Kidney *P* value	Lung *P* value	Liver *P* value	Spleen *P* value
**Pla2G6$^{+/+}$ vs. Pla2G6$^{+/+}$ 1/10 dose**	> 0,9999	> 0,9999	> 0,9999	> 0,9999	> 0,9999	> 0,9999
**Pla2G6$^{+/+}$ vs. Pla2G6$^{+/+}$ 1/2 dose**	0.339	> 0,9999	> 0,9999	0.2685	0.639	> 0,9999
**Pla2G6$^{+/+}$ vs. Pla2G6$^{+/+}$ Full-dose**	> 0,9999	> 0,9999	> 0,9999	> 0,9999	0.9247	0.339

	Total WBC *P* value	Neutrophils *P* value	Lymphocytes *P* value	Monocytes *P* value	Eosinophils *P* value
**Pla2G6$^{+/+}$ vs. Pla2G6$^{-/-}$ Sema**	0.1143	0.4857	0.6571	0.0286	0.3429

	Basophils *P* value	Haematocrit *P* value	Platelets *P* value	Red blood cells *P* value	Haemoglobin *P* value
**Pla2G6$^{+/+}$ vs. Pla2G6$^{-/-}$ Sema**	0.3429	0.0286	0.6571	0.0286	0.0286

Table S10. P values for Fig. S1.

Total distance (m)

	W9 *P* value	W12 *P* value
**Pla2G6$^{+/+}$ vs. Pla2G6$^{-/-}$**	0.0843	0.0013
**Pla2G6$^{+/+}$ vs. Pla2G6$^{-/-}$ Sema**	0.9975	0.0563
**Pla2G6$^{-/-}$ vs. Pla2G6$^{-/-}$ Sema**	0.1721	0.3991

Immobility time (s)

	W9 *P* value	W12 *P* value
**Pla2G6$^{+/+}$ vs. Pla2G6$^{-/-}$**	0.0485	0.0027
**Pla2G6$^{+/+}$ vs. Pla2G6$^{-/-}$ Sema**	0.9529	0.0463
**Pla2G6$^{-/-}$ vs. Pla2G6$^{-/-}$ Sema**	0.1845	0.6291

Mean speed (m.s-1)

	W9 *P* value	W12 *P* value
**Pla2G6$^{+/+}$ vs. Pla2G6$^{-/-}$**	0.0895	0.0016
**Pla2G6$^{+/+}$ vs. Pla2G6$^{-/-}$ Sema**	0.9978	0.074
**Pla2G6$^{-/-}$ vs. Pla2G6$^{-/-}$ Sema**	0.1779	0.3783

Max speed

	W9 *P* value	W12 *P* value
**Pla2G6$^{+/+}$ vs. Pla2G6$^{-/-}$**	0.6881	0.2067
**Pla2G6$^{+/+}$ vs. Pla2G6$^{-/-}$ Sema**	0.9891	0.8286
**Pla2G6$^{-/-}$ vs. Pla2G6$^{-/-}$ Sema**	0.8845	0.0707

Mobility time (s)

	W9 *P* value	W12 *P* value
**Pla2G6$^{+/+}$ vs. Pla2G6$^{-/-}$**	0.0366	0.001
**Pla2G6$^{+/+}$ vs. Pla2G6$^{-/-}$ Sema**	0.9462	0.0245
**Pla2G6$^{-/-}$ vs. Pla2G6$^{-/-}$ Sema**	0.1544	0.5521

	W9 *P* value	W14 *P* value
**Pla2G6$^{+/+}$ vs. Pla2G6$^{-/-}$**	0.5659	< 0,0001
**Pla2G6$^{+/+}$ vs. Pla2G6$^{-/-}$ Sema**	0.9988	0.2048
**Pla2G6$^{-/-}$ vs. Pla2G6$^{-/-}$ Sema**	0.7142	0.0151

Table S11. P values for Fig. S3.

	Ct Values GAPDH						
	GLP1-R *P* value	ATF-3 *P* value	GSK3β *P* value	CREB *P* value	BClxL *P* value	Bcl2 *P* value	Bax *P* value
**Pla2G6$^{+/+}$ vs. Pla2G6$^{-/-}$**	0.7375	0.9426	0.7581	0.78	0.264	0.841	0.999
**Pla2G6$^{+/+}$ vs. Pla2G6$^{-/-}$ Sema**	0.9221	0.658	0.8479	0.6105	0.4353	0.3789	0.9543
**Pla2G6$^{-/-}$ vs. Pla2G6$^{-/-}$ Sema**	0.9284	0.8438	0.4383	0.9554	0.9261	0.6981	0.9665
